# Exposure of a specific pleioform of multifunctional glyceraldehyde 3-phosphate dehydrogenase initiates CD14-dependent clearance of apoptotic cells

**DOI:** 10.1038/s41419-021-04168-8

**Published:** 2021-09-30

**Authors:** Surbhi Chaudhary, Anil Patidar, Asmita Dhiman, Gaurav Kumar Chaubey, Rahul Dilawari, Sharmila Talukdar, Radheshyam Modanwal, Manoj Raje

**Affiliations:** Institute of Microbial Technology, CSIR, Sector 39A, Chandigarh, 160036 India

**Keywords:** Cell biology, Apoptosis

## Abstract

Rapid clearance of apoptotic cells by phagocytes is crucial for organogenesis, tissue homeostasis, and resolution of inflammation. This process is initiated by surface exposure of various ‘*eat me’* ligands. Though phosphatidylserine (PS) is the best recognized general recognition ligand till date, recent studies have shown that PS by itself is not sufficient for clearance of apoptotic cells. In this study, we have identified a specific pleioform of GAPDH (Glyceraldehyde 3-phosphate dehydrogenase) that functions as an ‘*eat me’* signal on apoptotic cell surface. This specific form of GAPDH which is exposed on surface of apoptotic cells was found to interact with CD14 present on plasma membrane of phagocytes leading to their engulfment. This is the first study demonstrating the novel interaction between multifunctional GAPDH and the phagocytic receptor CD14 resulting in apoptotic cell clearance (efferocytosis).

## Introduction

Apoptotic cell removal is crucial for maintaining homeostasis in living organisms. On an average, the human body turns over 2–3 × 10^9^ cells each day. This turnover is vital not only in embryonic and postnatal development but also for routine tissue homeostasis. The process of apoptotic cell clearance by professional (macrophages and dendritic cells) and non-professional phagocytes (fibroblast, epithelial, endothelial cells) is termed as efferocytosis. Failure to remove apoptotic cells leads to secondary necrosis. It is vital that this process of cellular elimination remains immunologically quiescent and not involve any inflammatory response as this would generate an immune response against self antigens leading to inflammatory and autoimmune diseases [1–4]. Efferocytosis is a coordinated orchestration of multiple sequential events involving recognition, binding, ingestion, and digestion of apoptotic cells by phagocytes. This involves diverse interrelated signals. First, dying cells release ‘*find me’* signals (such as UTP, sphingosine-1-phosphate, ATP, thrombospondin-1, CX3CL1, and lysophosphatidylcholine) to attract or to activate phagocytes to the site of cell death [5, 6]. Subsequently, apoptotic cells expose *‘eat me’* signals on their surface. The recruited macrophages then utilize specific receptors which can recognize a dying cell among a population of viable cells. One of the crucial *‘eat me’* markers identified is phosphatidylserine [7]. Some others which have been identified include ICAM-3, calreticulin, annexin 1, and complement C1q [8–10]. The counterparts for these markers on phagocytes are known as *phagocytic receptors* and include T-cell immunoglobulin, mucin-domain-containing molecule (Tim-4), stabilin-2, and brain-specific angiogenesis inhibitor 1(BAI1) all of which have been reported to directly bind PS. CD14 is another well-recognized phagocytic receptor but its exact ligand has so far remained unknown [11]. Though phosphatidylserine exposure is known to be necessary for efferocytosis there is considerable ambiguity if, only phosphatidylserine exposure by itself is adequate to trigger engulfment of apoptotic cells. There exists considerable evidence that viable cells can also expose phosphatidylserine on their outer leaflet to the same extent as apoptotic cells but are not ingested by macrophages [12]. Consequently, there should be some additional factors involved in the efficient clearance of apoptotic cells that supplement PS exposure. Previously, the existence of certain other evolutionarily conserved protein determinants that are presented on the surface of dying cells has been reported. These have been termed as ‘SUPER’ (surface exposed during apoptotic cell death, ubiquitously expressed, protease sensitive, evolutionarily conserved, and resident normally on viable cells). Among these, certain glycolytic enzymes are believed to represent conserved biomarkers of apoptosis though their exact physiological role in the process of cell clearance has remained unclear [13]. GAPDH is a key enzyme involved in glycolysis and it is ubiquitously present in nature in all species ranging from bacteria to humans and is found predominantly in the cytosol. Several studies have revealed that GAPDH exhibits diverse multiple functions including a role in cancer and neuronal degenerative diseases [14, 15]. Switching between specific post translationally modified forms (plieoforms) has been reported to be one of the mechanisms by which GAPDH is able to switch between its myriad functions [16, 17]. Earlier our laboratory has demonstrated that mammalian cells express GAPDH on their surface, where it can act as a receptor for the iron carrier proteins transferrin and lactoferrin [18, 19]. In the current study, while testing the hypothesis regarding the role of GAPDH in apoptotic cell clearance via efferocytosis, we demonstrate that a pleioform of GAPDH enacts the role of an ‘*eat me’* signal on apoptotic cells, resulting in their internalization by phagocytes. GAPDH functions as a ligand on apoptotic cells and interacts with phagocytic receptor CD14 on macrophages enabling their clearance. Our investigations establish a novel moonlighting role of GAPDH in efferocytosis.

## Results

### Surface expression of GAPDH on apoptotic cells

**Fig. 1:**
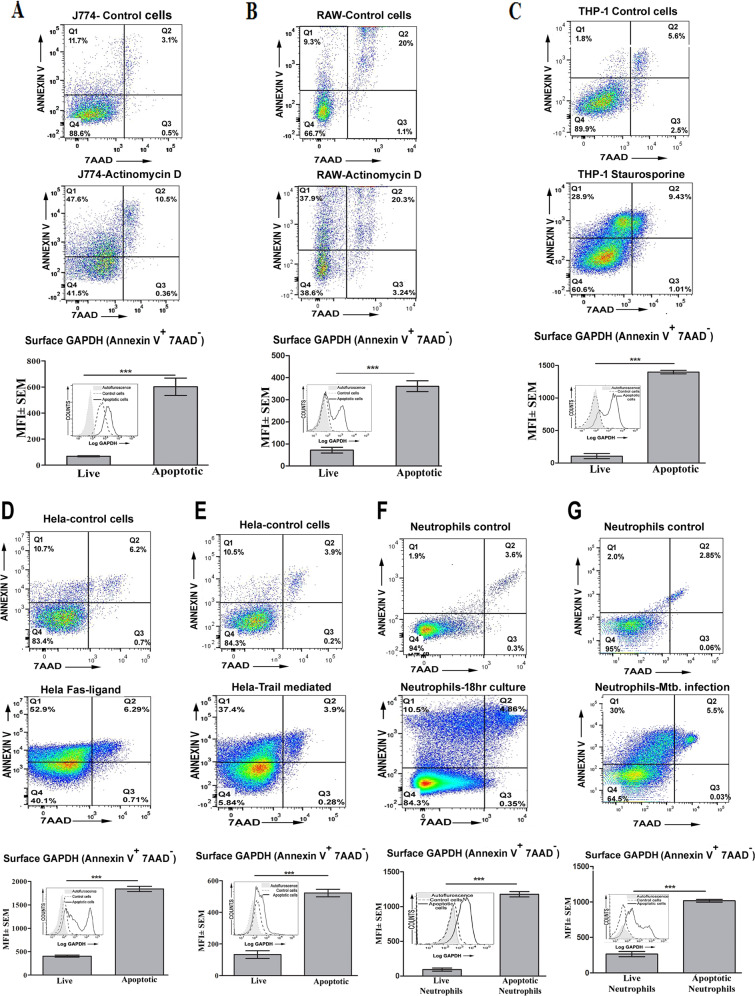
Apoptotic cells demonstrate elevated exposure of GAPDH as compared to control cells. Flow cytometric analysis of cells induced to undergo apoptosis by the intrinsic pathway using actinomycin D treatment; J774 cells (**A**), RAW cells (**B**), and staurosporine-treated THP-1-derived macrophages (**C**). Hela cells were induced to undergo apoptosis by the extrinsic pathway via Fas ligand (**D**) and TRAIL (**E**). Apoptosis was induced in, mouse neutrophils under physiological conditions (**F**) or due to infection with *M.tb* bacilli (**G**). Early apoptotic cells were identified by PS exposure, i.e., cells that stain +ve with annexin V-FITC but –ve with 7AAD (upper left quadrant in dot plot panels). GAPDH signal was recorded from these cells by applying gate in flow cytometry-based analysis. Data in bar graphs and overlay histograms (inset of bar graph) are from representative experiments and presented as mean fluorescence intensity (MFI) of surface GAPDH on live and early apoptotic cells (annexin V-FITC + ve but 7AAD–ve) and log fluorescence intensity of surface GAPDH signal from control and apoptotic cells (***P < .0001, *n* = 10^4^ cells). All experiments were repeated independently at least three times.

Earlier reports have indicated that GAPDH expression is enhanced on surface of some cell lines induced to undergo apoptosis. To test if this process was independent of the pathway by which apoptosis was induced in cells, we induced apoptosis via; (i) the intrinsic pathway by treatment with actinomycin D and staurosporine and also by (ii) the extrinsic pathway through Fas ligand and TRAIL-mediated initiation. We observed that apoptotic cells (staining positive for annexin V but negative for 7AAD) expose significantly more GAPDH on their surface as compared to healthy control cells (Fig. 1A–E). The cells were also confirmed to have initiated caspase activation ( Fig S1A–C and S2A). We also found an increase in surface GAPDH expression of primary neutrophils that were undergoing apoptosis under physiological conditions or after infection with Mycobacterium tuberculosis (H37*Rv*) (Fig. 1F-G). These results and the reports of other groups indicate that, regardless of the pathway of induction, cells undergoing apoptosis expose more GAPDH on their surface.

### GAPDH recruited to apoptotic cell plasma membrane is an isoform distinct from those recruited during other physiological stresses

**Fig. 2:**
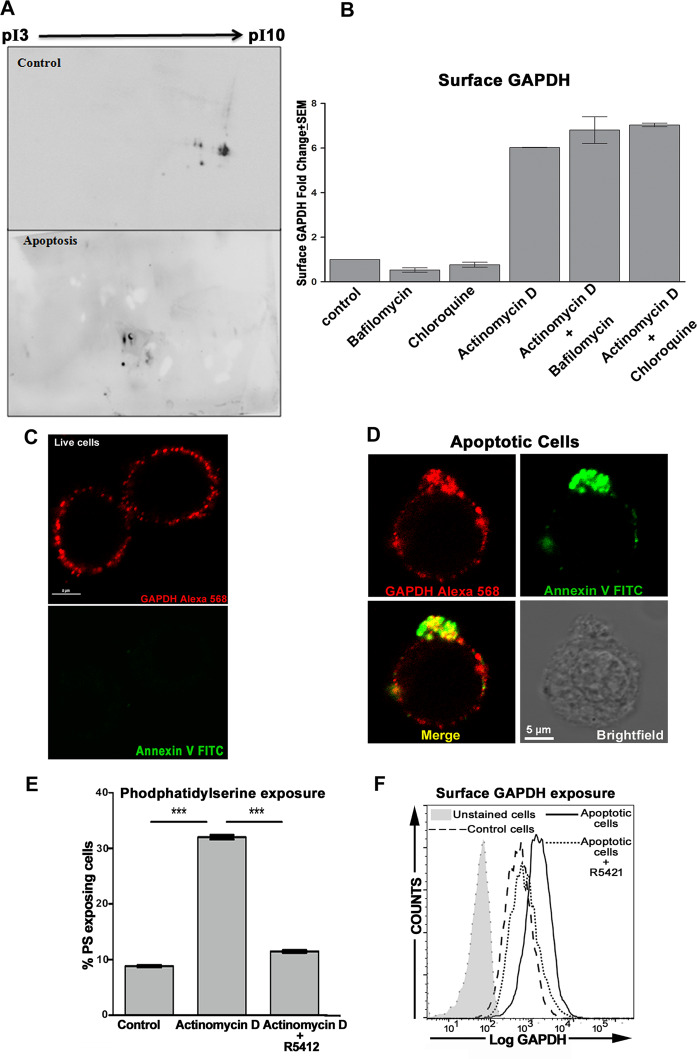
GAPDH exposed on apoptotic cells is a distinct isoform and is mobilized by a scramblase sensitive mechanism. **A** Western blot of 2D-gel-electrophoresis separated GAPDH isoforms from membrane fractions of untreated control and apoptotic J774 cells, pI: isoelectric point. **B** Exposure of GAPDH on J774 cell surface is not sensitive to inhibitors of lysosomal exocytosis. **C**, **D** Distribution of GAPDH (red) and phosphatidylserine (green) on J774 cell membrane. GAPDH demonstrates a punctuate distribution on surface of live cells which are devoid of PS staining (**C**). On apoptotic cells the two signals co-localize in patches and cap formations (**D**). Scale bar 5 µm. **E** Inhibition of scramblase significantly decreases PS exposure on cells induced to undergo apoptosis. Graph presents the percentage of J774 cells responding with PS exposure (annexin V-FITC positive cells) after treatment with actinomycin D (2 μg/ml) for 6 h in the absence or presence of the scramblase inhibitor R5421 (100 μM). PS exposure was evaluated by florescence staining and flow cytometry, see Appendix Fig. S1F for 2-D flow cytometry plots. **F** Cell surface GAPDH exposure on J774 cells is also significantly diminished by scramblase inhibition. Data in overlay histogram plot is from representative of three experiments.

The existence of, discrete post-translationally modified isoforms (pleioforms) of GAPDH, performing independent functional activities is known [20]. A switching of GAPDH in membrane of bone-marrow-derived macrophages, upon iron loading to a more alkaline isoform, has been reported earlier [21]. Cellular homeostatic processes like iron depletion or intracellular iron loading have been shown to recruit significantly additional and distinct forms of GAPDH onto cell surface that bind either holo-transferrin or apo-transferrin, respectively [16, 19]. We observed that, unlike the GAPDH recruited upon modulation of cellular iron, the GAPDH recruited upon induction of apoptosis does not result in any significant increase of cellular binding by either holo or apo transferrin (Fig. S2B & C). Significantly, the additional GAPDH recruited onto cell surface due to modulation of cellular iron (Fig. S2D) does not induce any increase in engulfment of the cells by macrophages (Fig. S2E). Analysis of apoptotic cell membrane protein fractions by two-dimensional (2D) gel electrophoresis and Western blotting revealed an acidic shift in the predominant GAPDH isoforms of apoptotic cells as compared with those from control cells (Fig. 2A). They were also different from our earlier reported GAPDH isoforms observed on iron loaded as well as iron-depleted cells [16]. Liquid chromatography–tandem mass spectrometry (LCMS/MS) analysis of membrane-associated GAPDH from apoptotic cells revealed a higher abundance of various post-translational modifications (PTMs), including oxidation, dimethylation, acetylation, deamidation, myristoylation, succinyl lysine, ADP ribosylation, proline oxidation to pyroglutamic acid and palmitoylation as compared to GAPDH from the membranes of control live cells (Table S1).

### GAPDH recruitment to plasma membrane upon apoptosis is not sensitive to inhibitors of lysosomal exocytosis, but is associated with PS exposure and is sensitive to scramblase inhibition

**Fig. 3:**
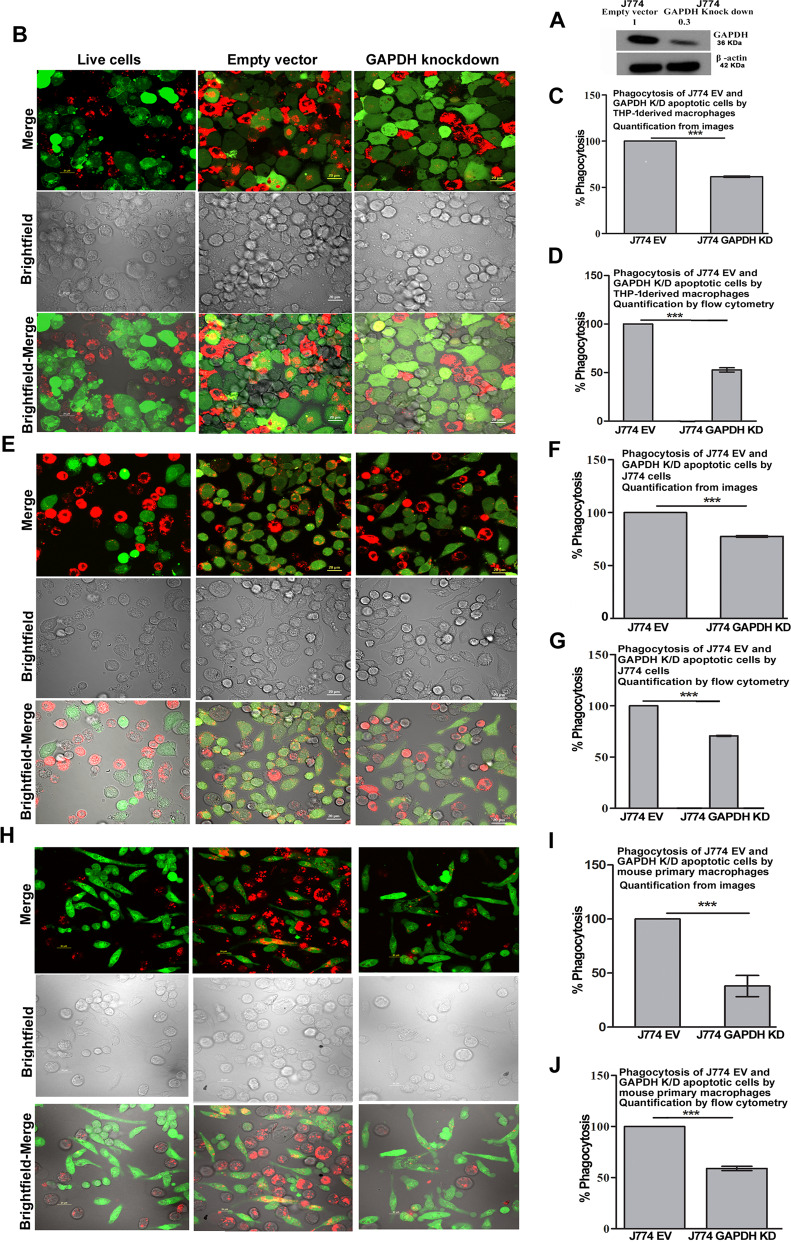
GAPDH exposure upon apoptosis facilitates phagocytosis. **A** Knock down of GAPDH. Western blot of cell lysates from empty vector control and GAPDH K/D J774 cells. **B** Representative confocal images of live and apoptotic J774 cells (red color) phagocytosed by THP-1 cells (green). Scale bar, 20 μm. **C** Quantification from random confocal images to evaluate magnitude of phagocytosis, graph is presented as percentage of phagocytosis ± SD of apoptotic J774 cells where GAPDH has been knocked down and compared with phagocytosis of apoptotic empty vector transfected controls. Images are representative of results obtained from 3 independent experiments (***P < 0.0001, *n* = 200 cells). **D** Flow cytometry-based analysis to determine the percentage of THP1 phagocytes that had phagocytosed J774 apoptotic cells. Data is represented as % phagocytosis ± SD, taking phagocytosis of J774 empty vector apoptotic cells as 100%. Experiment was repeated three times (***P < 0.0001, *n* = 3). **E** Representative confocal images showing phagocytosis of live and apoptotic J774 cells (red color) with wild-type J774 cells performing the role of phagocytes (green). **F** Quantification of phagocytosis from confocal images obtained from three independent experiments, scale bar, 20 μm, (***P < 0.0001, *n* = 200 cells). **G** Flowcytometry analysis of percentage phagocytosis of J774 GAPDH K/D and empty vector control cells by J774 phagocytes. Data is represented as ± SD, taking phagocytosis of J774 empty vector apoptotic cells as 100%. Experiment was repeated three times (***P < 0.0001, *n* = 3). **H** Representative confocal images of phagocytosis of live and apoptotic J774 cells (red color) by peritoneal macrophages (green). **I** Quantification of phagocytosis from confocal images obtained from three independent experiments, scale bar, 20 μm, (***P < 0.0001, *n* = 100 cells). **J** Flow cytometry-based analysis of apoptotic J774 GAPDH K/D and empty vector transfected cell phagocytosis by peritoneal macrophages. Data is represented as ± SD taking phagocytosis of J774 empty vector apoptotic cells as 100% (***P < 0.0001, *n* = 3).

Trafficking of GAPDH to cell surface has recently been demonstrated to be via the non classical secretory pathway of lysosomal exocytosis [22]. Inhibition of this pathway in cells induced to undergo apoptosis had no effect on GAPDH exposure (Fig. 2B). GAPDH on surface of live cells was observed to be present in a ring of punctae all over the membrane (Fig. 2C), in contrast apoptotic cell surface GAPDH was observed to concentrate into large patch and cap-like formations where it co-localized with the apoptotic cell marker phosphatidylserine (Fig. 2D). Such a distribution has been reported earlier in case of other ‘*eat me’* signals [8]. Induction of apoptosis is known to activate phospholipid scramblases which are responsible for externalization of phosphatidylserine from inner to outer leaflet of plasma membrane [23]. As GAPDH is known to interact with phosphatidylserine on the cytosolic side of the cells [24] we hypothesized that this inner leaflet GAPDH is recruited onto the cell surface of apoptotic cells along with the scramblase-induced tumbling of PS. Ethaninidothioic acid (R5421) has been used as a scramblase inhibitor to determine the role of phospholipid scrambling across a range of systems and is known to inhibit the externalization of PS on surface of apoptotic cells [25, 26]. Treatment of J774 cells, that had been induced to undergo apoptosis, with R5421 caused a significant decrease in percentage of cells exposing PS as compared to control apoptotic cells (only actinomycin D treated), this correlated with a significant decrease in exposure of GAPDH (Fig. 2E, F and Fig. S2F). That R5421 inhibits only scrambalses leading to decrease in PS exposure and not the entire process of apoptosis was confirmed by a caspase 3 assay (Fig. S2G). GAPDH exposure on apoptotic cells is vital for their clearance (Efferocytosis). To ascertain if GAPDH that is recruited onto apoptotic cell surface facilitates in their clearance by efferocytosis, we first performed a knockdown of GAPDH in target cells (Fig. 3A) and confirmed that in both, KD and empty vector control cell populations, the percentage of apoptotic cells was comparable (Fig. S2H). However, GAPDH K/D apoptotic cells exposed significantly lower amount of GAPDH on their surface as compared to empty vector transfected control cells (Fig. S2I, J). These cell lines were then used for efferocytosis by THP-1 phagocytes. Significantly less phagocytosis of GAPDH KD apoptotic cells as compared to their J774 empty vector control counterparts was recorded by both, confocal microscopy-based quantification (Fig. 3B, C) and also by flowcytometry (Fig. 3D). Similar results were obtained when the THP-1 cells were replaced with either J774 wild-type cells (Fig. 3E–G) or primary mouse peritoneal macrophages (Fig. 3H–J) to perform the role of phagocytes. Since GAPDH knockdown and empty vector cells both undergo apoptosis to the same extent and demonstrate no difference in PS exposure (Fig. S2H) the observed decrease in phagocytosis could be attributed to deficit of GAPDH exposure on apoptotic cells thereby implicating this moonlighting molecule in efferocytosis.

### Apoptotic cell surface GAPDH interacts with phagocyte membrane CD14 and is responsible for efferocytosis

**Fig. 4:**
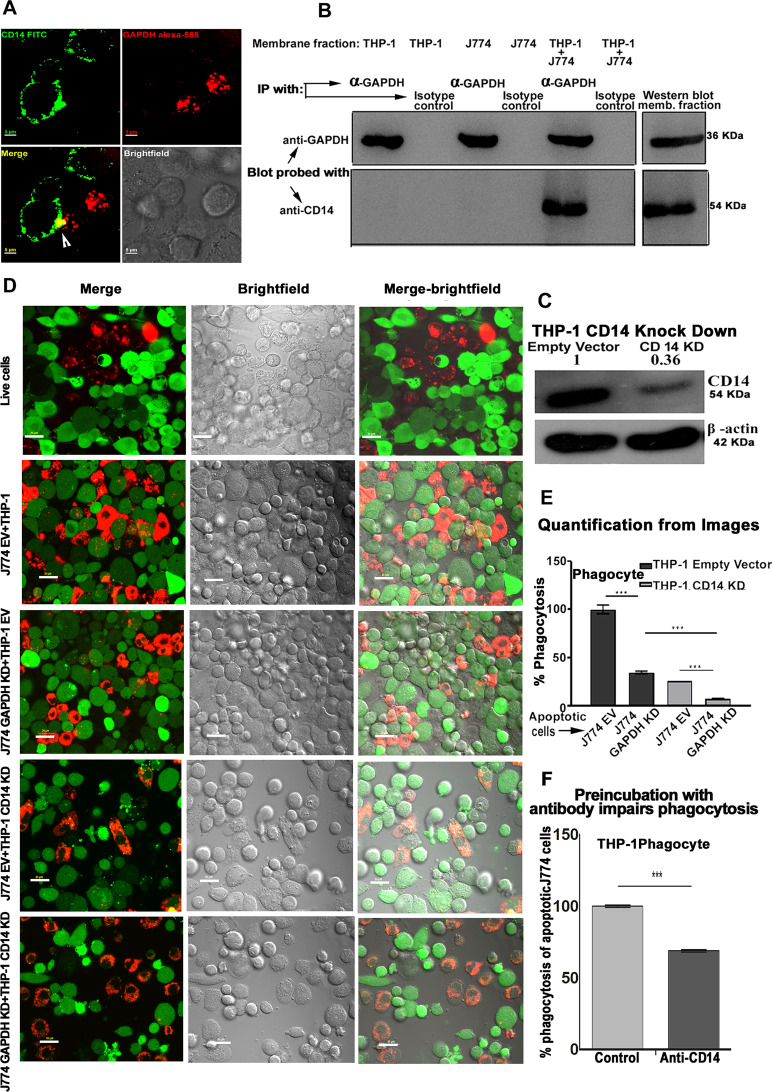
GAPDH exposed on apoptotic cells and phagocyte CD14 mediate efferocytosis. **A** Co-localization of apoptotic cell surface GAPDH with CD14 on phagocyte. When cells were incubated together the two signals co-localize at points of intercellular contact. Scale bar, 5 μm. **B** Co-immunoprecipitation (Co-IP) of GAPDH from mixed membrane fractions of apoptotic cells (J774) and phagocytes (THP-1). Right side panels are Western blots of membrane fractions as positive control for both antibodies. **C** Western blot to confirm the CD14 K/D in THP-1 cells. Cell lysates were prepared from THP-1 empty vector and CD14 knockdown cells and samples were run on 10% SDS-PAGE. Separated proteins were transblotted onto PVDF membrane and probed with mouse anti-CD14 antibody (Abcam) followed by secondary antibody anti-mouse peroxidase (Sigma). **D**–**E** Phagocytosis of apoptotic cells is dependent upon both GAPDH on apoptotic cell surface and CD14 on phagocytes. **D** Representative confocal microscopy images of live and apoptotic J774 cells that were phagocytosed by THP-1 phagocytes. Live cells, apoptotic empty vector, and GAPDH K/D J774 cells are labeled with Vybrant DiD dye (Red). Phagocyte THP-1 cells, empty vector, and CD14 K/D cells are labeled with CFSE (Green). Scale bar, 20 μm. **E** Bar graph represents phagocytosis as percentage of control (extent of phagocytosis by THP-1 empty vector cells incubated with J774 empty vector cells as 100%), ***P < 0.0001, *n* = 150 cells, also see Fig. S2C–D. **F** Disruption of the GAPDH–CD14 interaction utilizing a blocking antibody against CD14 results in a significant decrease in phagocytes ability to engulf apoptotic cells. This corroborates and complements the results of our knockdown studies.

Earlier investigations have reported that macrophage CD14 functions as a phagocytic receptor facilitating efferocytosis [27]; however, the ligand for CD14 on apoptotic cells has remained unidentified [11, 28].Though interactions of CD14 with several multifunctional glycolytic proteins like alpha enolase have been documented earlier [29], there are no reports regarding its interaction with GAPDH which is a highly versatile and multifunctional molecule. Antibody staining and confocal microscopy-based observations of co-cultured cells indicated that GAPDH and CD14 from two interacting cells can co-localize (Fig. 4A). Utilizing a mixture of apoptotic cell and phagocyte membranes the interaction between phagocyte membrane CD14 with GAPDH exposed on surface of apoptotic cells was confirmed by co-immunoprecipitation. At the same time no interaction between phagocyte CD14 with GAPDH from its own membrane could be detected (Fig. 4B). In addition, when the J774 apoptotic cell fraction was replaced with membrane from live cells, no significant interaction was observed (Fig S3A). To determine if, interaction between apoptotic cell surface GAPDH and phagocyte CD14, can play any role in their clearance we prepared a THP-1 CD14 knockdown cell line along with its empty vector control (Fig. 4C). These cells, deficient in their surface CD14 expression (Fig. S3B) were utilized for phagocytosis of apoptotic J774 GAPDH knockdown and J774 empty vector cells. We observed a significant decrease in phagocytosis when either only ligand i.e., GAPDH or only receptor i.e., CD14 knock down cells were utilized as compared to results of parallel experiments where empty vector control cell lines were utilized. In case where both the ligand (GAPDH) as well as receptor (CD14) was knocked down, a synergistic effect of reduction in phagocytosis could be observed by both, confocal microscopy (Fig. 4D, E) as well as by flow cytometry (Fig. S3C, D) based assays of phagocytosis. Blocking of phagocyte CD14 with antibody also resulted in a significant inhibition of phagocytosis (Fig. 4F).

## Discussion

Timely and regulated clearance of deceased individuals in any population is essential for the maintenance of a healthy & robust society, organ or tissue. Just as in human society this involves an orchestrated set of events involving substantial interaction between undertakers and the corpse. At the cellular level too, the foremost requirement for efficient clearance of apoptotic cells is a physical interaction between apoptotic cell and phagocyte [30]. This is achieved by the recognition and interaction of exposed *‘eat me’* signals on apoptotic cells and phagocytic receptors on phagocytes [31–33]. In the current study, we have identified GAPDH as a novel ligand or *‘eat me*’ signal crucial for recognition and clearance of apoptotic cells. Though GAPDH is predominantly a cytosolic protein, recruitment of this multifunctional molecule to the cell surface has been observed to occur in several altered physiological states in live cells. Earlier studies have revealed different pleioforms of GAPDH being presented on membrane which are utilized for specific purposes. Iron depleted and iron overloaded cells expose GAPDH that can bind to holo or apo forms of transferrin, respectively. These cells are otherwise healthy, can proliferate, and do not expose PS. While the former serves to supply the metal to iron deficient cells the latter removes excess iron from overloaded cells [16, 19, 34, 35]. In the current study GAPDH expression was found to be increased on surface of apoptotic cells. This is in agreement with the reports by earlier investigators [13, 36]. Our 2D gel electrophoresis studies revealed a marked acidic shift in the isoelectric point of GAPDH on apoptotic cell surface indicating that perhaps a specific peioform was being recruited for a particular function. Similar shift in isoelectric point of proteins in apoptosis have been reported previously [13]. LC/MS–MS analysis revealed that in comparison to GAPDH present on control viable cell surface, the GAPDH recruited after apoptosis has a higher abundance of several PTM’s. These modifications are known to shift the isoelectric point of proteins as observed by us and also reported previously [37–39]. Post-translational myristoylation, acetylation, deamidation, succinylation, ADP ribosylation of other proteins in apoptosis has been well reported by numerous researchers [40–46]. Though GAPDH is also present on viable cells, its patchy distribution and upregulation on surface of apoptotic cells along with certain PTM’s can explain the importance of apoptotic cells surface GAPDH as an “eat me signal”. However further characterization of this specific pleiform of GAPDH may be needed to fully explain its detailed role in cellular clearance. A recent study has highlighted the role of mammalian cell surface recruited GAPDH in host pathogen interaction. Host GAPDH on surface of dying lung epithelial cells was found to interact with *Strepococcus pneumoniae* resulting in secondary infection following influenza infection [47]. This study demonstrated that endogenous GAPDH was recruited onto the surface of dying lung epithelial cells which had been infected with either, Influenza A virus (IAV) or *Streptococcus pneumoniae*. This host GAPDH was then observed to interact with the Pneumoccocal surface protein A (PspA) and facilitate the entry of bacteria into the IAV infected cells thereby exacerbating the damage caused by IAV. In our current study, as an, eat me ligand, the exposed GAPDH interacts with CD14 (phagocytic receptor) present on phagocyte membrane. CD14 is a pattern recognition receptor (PRR) that has been shown to interact with endotoxin LPS and generate inflammatory response [48]. Apart from a role in promoting inflammation, in vitro and in vivo studies have shown that membrane CD14 also plays a significant role in clearance of apoptotic cells [27, 49, 50]. However, this CD14-dependent engulfment of apoptotic cells is an anti-inflammatory response by macrophages. The ligand to which CD14 interacts on apoptotic cells has remained unknown till date [28]. A recent study on efferocytosis of SARS-CoV-2-infected cells found impairment in the anti- inflammatory response by phagocytes due to reduction in CD14 expressing anti-inflammatory monocytes that execute efferocytosis more efficiently [51]. In our current study, we have identified for the first time that a specific post translationally modified form of GAPDH recruited to the surface of apoptotic cells interacts with phagocyte CD14. We also confirmed that CD14 does not interact with GAPDH present on viable cells. Knockdown studies of CD14 and GAPDH confirms the role of GAPDH–interaction in efferocytosis. An earlier study by our group had revealed that, GAPDH is trafficked to the surface of cells via the non classical pathway of lysosomal exocytosis [22]; however, our current investigations have shown that inhibition of this pathway does not have any effect on the GAPDH exposure on apoptotic cells. Interestingly, we observed that GAPDH is externalized on surface of apoptotic cells through a scramblase-dependent mechanism as in the case of PS exposure. Recently, Cheshenko and coworkers [52] have reported that phospholipid scramblase-1, a calcium responsive enzyme that flips PS from the inner to outer leaflet of the plasma membrane can also concurrently bring about externalization of Akt and possibly other inner leaflet proteins. However, further studies are required to fully understand mechanistic details of this process and the scramblases involved [53, 54].

Our current study provides a novel insight in understanding the unidentified mechanism of efferocytosis involving a moonlighting function of the glycolytic enzyme GAPDH and CD14. A limitation of working with GAPDH is that, being a housekeeping molecule, there are significant challenges in extending the work for in-vivo studies as generating a GAPDH knockdown or knockout animal can be lethal. In future investigations it would be important to study the mechanism of GAPDH–CD14 interaction at the molecular level, understand its effects on intracellular signaling in phagocytes and identify the signaling intermediates involved. Rac1 is a pleiotropic regulator of many cellular processes belonging to the family of Rho-GTPases and is involved in the signaling of engulfment of apoptotic cells [55, 56]. In future it could be worthwhile to explore its role in CD14-dependent efferocytosis. Efferocytosis is a crucial cellular clearance process essential to maintain homeostatic balance in organisms and by studying the molecular mechanism of apoptotic cell clearance via moonlighting GAPDH we hope to provide a new insight for understanding the inflammatory disease processes associated with efferocytosis [57–61].

## Methods and materials

### Cell lines, primary cells, and materials

J774 (mouse macrophage cell line) was procured from ECACC and maintained in DMEM high glucose with 10% fetal calf serum (FCS). The CHO-TRVb, (derived from CHO) cell line which lacks both TfR1 & 2 was kindly provided by Prof. Timothy McGraw and has been described by us earlier for analysis of transferrin binding to cell surface GAPDH [19]. All other cell lines were obtained from National Centre for Cell Sciences, Pune, India. THP1 cells were activated into macrophages by 24 h incubation with 25 ng/ml phorbol-12-myristate-13-acetate (PMA) and maintained in RPMI-1640 medium supplemented with 10% FCS. Peritoneal macrophages were purified as described previously from C57BL/6mice [62, 63]. Neutrophils were isolated by histopaque (sigma)-based density gradient centrifugation [64]. Use of mice as a source of primary neutrophils and all animal handling protocols were approved by the statutory animal ethics committee. Annexin V-FITC was obtained from BD Biosciences. CellTrace™ CFSE Cell Proliferation Kit, Vybrant cell-labeling solutions (DID, DIO, DIL). Luminata Forte Western HRP substrate was obtained from Merck Millipore. All kits and dyes were utilized as per manufacturer’s instructions. Modulation of cellular iron was as per previously published protocols [16]. Briefly cellular iron depletion was achieved by culturing cells for 24 h in medium containing 100 μm Desferrioxamine (DFO) and to load cells with iron the medium was supplemented with 100 μm FeCl_3_. Lysosomal exocytosis was inhibited using incubation with bafilomycin or chloroquine exactly as described previously [22].

### Induction of apoptosis

J774 cells were induced to undergo apoptosis by incubation for 6 h at 37 °C, with 2 µg/ml actinomycin D, RAW cells were induced using 1 µg/ml actinomycin D for 4 h. THP-1 cells were induced to undergo apoptosis by incubation with 1 µg/ml of staurosporine (calbiochem) for 12 h in culture. Hela cells were induced to undergo apoptosis by 300 ng/ml Fas ligand (sigma) for 24 h and 300 ng/ml TRAIL (sigma) for 12 h in complete RPMI. Neutrophils were induced to undergo apoptosis under physiological conditions by culturing them in complete RPMI for 18–20 h [65] and also by infection with *Mycobacterium tuberculosis* (*M.tb H37Rv*) as described previously [66, 67]. Briefly cells were incubated with *M.tb* bacilli for 30 mins and then cultured for 18 h in fresh medium. In all cases apoptosis was confirmed by detecting PS exposure on cell membrane using Annexin V staining while necrotic cells were identified as those staining positive with 7-aminoactinomycin D (7AAD, Invitrogen #A1310) using flow cytometry as described below.

### Flow cytometery-based evaluation of apoptosis, cell surface GAPDH exposure, and binding of transferrin

To confirm phosphatidylserine exposure, aliquots of 5 × 10^5^ cells were washed twice with PBS and then resuspended in 100 ul of annexin V binding buffer (10 mM HEPES (pH 7.4), 150 mM NaCl, and 2.5 mM CaCl_2_) and incubated with 5 µl of annexin V-FITC conjugate. Maintenance of plasma membrane integrity was evaluated by counter staining with 7-AAD. Cell surface GAPDH recruitment was analyzed exactly as described previously [16]. Briefly 5 × 10^5^ J774 cells were washed three times with FACS buffer [20 mM HEPES (pH 7.4), 150 mM NaCl, 1 mM CaCl_2_, 1 mM MgCl_2_, 5 mM KCl supplemented with 5% FCS] and blocked with FACS block (FACS buffer further supplemented with 5% each of normal goat serum and normal human serum). Subsequently cells were stained with rabbit anti-GAPDH antibody (Sigma #G9545) or isotype control (rabbit IgG, (Invitrogen#10500 C)) followed by goat anti-rabbit-IgG-alexa647 (Invitrogen#A-21244). Forward and side scattering signals were utilized to exclude cellular doublets and obtain fluorescent signal only from single cells. Analysis of GAPDH signal from apoptotic cells, (gated for staining annexin V + ve but 7AAD –ve), was done for each sample of apoptotic cells using a FACS Aria or FACS Verse flow cytometer (BD). GAPDH signal from live cells was acquired from cells staining negative for both 7AAD and annexin V. To evaluate any increase in binding of apo or holo transferrin to cells apoptosis was induced in CHO-TrVb cells with Actinomycin D, after confirming increased exposure of surface GAPDH (see Fig. S1D), the cells were incubated with either apo or holo transferrin that had been labeled with Alexa-647, and analyzed by flow cytometry. As positive control for increase in transferrin binding, CHO-TrVb cells which had either been iron depleted by incubation with iron chelator DFO or loaded with excess iron by supplementing the culture medium with FeCl_3_ were also stained with labeled transferrin.

### Phagocytosis assay

Assay to quantify phagocytosis was as described previously [68–71]. To evaluate phagocytosis of apoptotic cells, J774 cells that had been induced to undergo apoptosis were labeled with Vybrant DiD dye (exhibiting red fluorescence) and induction of apoptosis was confirmed as described above. Separately, phagocytes (THP-1 cells) were labeled with green fluorescent cell tracer dye CFSE and co-cultured with apoptotic J774 cells for 2 h in a ratio of 1:1 at 37 °C to allow for phagocytosis. Subsequently any residual surface-bound apoptotic cells were removed by wash with 5 mM EDTA in PBS. Next the cells were fixed in 2% paraformaldehyde and images were acquired on confocal microscope (Nikon A1R, Tokyo, Japan) using 60X oil emersion 1.49 numerical aperture objective lens with 1 AU aperture. The number of cells internalizing apoptotic cells was enumerated from random images. Macrophages which had ingested apoptotic cells were also analyzed by flow cytometry (as described above) to determine the percentage of their population ingesting apoptotic cells by observing the percentage of cells exhibiting both red and green signal i.e., cells having ingested apoptotic cells.

### Scramblase inhibition

R5421, a scramblase inhibitor described previously [26, 72] was obtained from Endotherm (Saarbruecken, Germany). It was dissolved to 100 mM concentration in DMSO as per manufacturer’s instructions. J774 cells were pre-incubated with 100 µm of R5421 for 1 h then treated with apoptosis inducer actinomycin D for 6 h. Then cells were further processed for externalization of phosphatidylserine and cell surface GAPDH exposure. Control cells were treated with only DMSO.

### Caspase assay

Activation of specific caspases was done using a CaspACE^TM^ assay system (Promega) exactly as per manufacturer’s instructions. Untreated control or actinomycin D/R5421-treated cells were harvested, washed with ice-cold PBS, and re-suspended in lysis buffer, and 50 μg of soluble proteins from each sample was incubated with colorimetric substrate at 37 °C for 4 h. Cleavage of substrate was analyzed by measuring absorbance at 405 nm.

### Co-localization of cell surface molecules

For co-localization of phosphatidylserine with GAPDH, apoptotic J774 cells were stained for surface GAPDH followed by annexin V-FITC at 4 °C. Immunostaining of cell surface proteins was performed essentially as described previously [16]. All cells were washed and fixed with 2% paraformaldehyde for imaging in confocal microscope as described above. To observe co-localization of CD14 on phagocytes with GAPDH on apoptotic cells, 5 × 10^5^ PMA activated THP-1 cells seeded in confocal dishes were stained with human anti-CD14-FITC antibody (BD bioscience #555397). Simultaneously 5 × 10^5^ J774 cells that had been induced to undergo apoptosis were stained for detection of surface GAPDH utilizing rabbit anti-GAPDH antibody followed by secondary goat anti-rabbit alexa-568.The THP-1 cells were co-incubated with apoptotic J774 cells at 4 °C for 1 h then for further 15 min at 37 °C before extensive washing to remove any unbound cells and imaging.

### Co-immunoprecipitation

Membrane fractions from THP-1 and apoptotic J774 cells were prepared as described previously [34, 62] and co-incubated on ice for 3 h. From this mixture GAPDH was immunoprecipitated using monoclonal mouse anti-GAPDH antibody (calbiochem #CB1001) immobilized onto anti-mouse-IgG-Magnabeads (Pierce). Co-IP of GAPDH was also performed using either only, THP-1 or J774 apoptotic cell membrane fractions. A set of negative controls was also performed in parallel, wherein the membrane fraction was attempted to be co-immunoprecipitated using isotype control mouse IgG (Invitrogen#10400 C) immobilized on anti-mouse-IgG–Magnabeads. All of the co-IP beads were boiled in SDS sample buffer, eluted proteins resolved on 10% SDS PAGE, transferred to nitrocellulose membrane and probed with either human anti-CD14antibody (Abcam #ab182032) or anti-GAPDH. As another control, similar experiments were performed where the membrane fraction of apoptotic J774 cells was replaced with a similar fraction from live cells.

### 2D analysis of membrane-associated GAPDH

2D analysis was performed as previously described [16]. Briefly, J774 cells were treated with 2 ug/ml of actinomycin D, and membrane fractions were prepared essentially as described earlier [34]. Membrane proteins were purified by using the Biorad Protein Cleanup Kit as per the manufacturer’s instructions and were subsequently dissolved in rehydration buffer (8 M urea, 7 M urea, 2% CHAPS, 50 mM dithiothreitol, 0.2%(w/v) biolyte 3/10 ampholytes and traces of bromophenol blue) to a final volume of 125 µl. IPG 7 cm, pH 3–10 linear gradient strips (BioRad) were loaded with samples by rehydration-loading. Isoelectric focusing was performed at 250 V for 20 min (linear), 4000 V for 2 h (linear), then maintained by 4000 V (rapid) for 2 h until 14000 V-hr was achieved. The current was limited to 50 µA per strip, and the temperature was kept at 20 °C for all isoelectric focusing steps. For the second dimension SDS-PAGE, the IPG strips were incubated in equilibration buffer 1 (6 M urea, 2% SDS, 20% glycerol, 0.375 M Tris-HCl pH 8.8, 2% DTT) for 10 min, followed by incubation in equilibration buffer 2 (6 M urea, 2% SDS, 20% glycerol, 0.375 M Tris-HCl pH 8.8, 2.5% iodoacetamide) for another 10 min and then transferred onto 4–15% gradient acrylamide gels (Biorad). The gels were run at 25 mA until the Bromophenol Blue front had reached the bottom of the gel. Resolved proteins were processed for Western blotting and immunodetection of GAPDH was done as described previously [34].

### In-gel digestion and peptide extraction

J774 cells were induced to undergo apoptosis by actinomycin D and then membrane fractions from apoptoic and control cell were purified. Membrane proteins (500 µg) from both samples were extracted, subjected to 12% SDS-PAGE, and stained with Coomassie Brilliant Blue. Bands corresponding to GAPDH were excised and sent to C-CAMP technology platform services (MS facility) Bengaluru for analysis. In gel digestion was as previously described [73]. Briefly, gel pieces were transferred into a microcentrifuge tube and 400 μL of destaining solution (100 mM ammonium bicarbonate/acetonitrile (1:1 vol/vol)) was added and mixed on a vortex mixer until the blue color eluted and gel became transparent. Subsequently, 400 μL of 100 mM acetonitrile was added and gel incubated at room temperature with occasional vortexing until it became white and shrunken. To remove acetonitrile trypsin buffer (13 ng/µL of trypsin in 10 mM ammonium bicarbonate containing 10% acetonitrile) was added to cover the dry gel pieces (100 µL) and kept at −20 °C for 2 h. Then 100 µL of ammonium bicarbonate buffer was added to gel pieces to wet them for enzymatic cleavage. The tubes were placed in a thermostat and incubated overnight at 37 °C. Then 5% formic acid/acetonitrile was added to the solution (twice the total volume of the solution) to stop the reaction. The solution was incubated for 15 min at 37 °C. The gel pieces were removed carefully and the supernatant was centrifuged for 20 min and dried. Residue was dissolved in 40 µL of 0.1% formic acid/2% acetonitril and used for LCMS analysis.

### Mass spectrometric analysis

Mass spectrometric analysis of the extracted peptides was performed at C-CAMP MS facility using EASY nLC 1200 coupled to LC precolumn (Pep map TM 100; 75 µm x 2 cm; Nanoviper C18, 3 µm; 100 Å) and LC analytical column (EASY SPRAY PEPMAP RSLC C18 3 µm; 15 cm × 75 µm; 100 Å). A sample volume of 5 µL was injected with the nanoflow rate of 300 nL/min. The mobile phase constituted 95% buffer A (0.1% Formic acid in HPLC water) and 5% of buffer B (80% Acetonitrile + 0.1% formic acid in HPLC water) for 5 min: 55% buffer A and 45% buffer B for 98 min: 5% buffer A and 95% buffer B for 102 min: 5% buffer A and 95% buffer B for 120 min. The MS/MS Scan Mode: FT-ICR/Orbitrap with scan range 100–2000 m/z. Ion Source used was ESI (nano-spray) with fragmentation mode: high energy CID (y and b ions). The raw files post-MS run were analyzed using PEAK as search engine in Uniprot database with following parameters: Parent Mass Error Tolerance: 10 ppm; Fragment Mass Error Tolerance: 0.6 Da; Precursor Mass Search: monoisotopic; Maximum Missed Cleavages: 2; Non-specific Cleavage: both; taxonomy was limited to Mus musculus; Fixed Modifications: Carbamidomethylation; variable modifications considered were: acetylation (K), deamidation (N, Q), methylation (D, E, S, T, K), dimethylation (N, R, K), oxidation (M, C), phosphorylation (S, T, Y), cysteine propionamide, conversion of praline to pyroglutamic acid (pyro-Glu; P), pyro-Glu (N-terminal, E, Q), nitrosylation (C), succinylation (K), ADP-ribosylation (K), palmitoylation (C, K, S, T), myristoylation (K, N-terminal, G), farnesylation (C) and GPI anchor (protein C-terminal). Only significant hits as defined by PEAK analysis were considered. A minimum peak peptide score (Peptide −10 lgP≥20); and protein score (protein −10 lgP≥20); De novo ALC Score ≥50%.

### Silencing of GAPDH and CD14

For cellular GAPDH and CD14 knockdowns, J774 cells were transfected with mouse GAPDH short hairpin RNA (shRNA) and THP-1 cells with human CD14 short hairpin RNA (shRNA) lentiviral particles (Sigma-Aldrich) as per manufacturer’s instructions. For controls separate sets of cells were transfected with pLKO.1-puro Non-target shRNA control lentiviral particles (Sigma-Aldrich). Stably transfected cells were selected and cultured in medium supplemented with puromycin (selection pressure of 7ug/ml). Knockdown was confirmed by Western blot and flow cytometry as described earlier [19].

### Disruption of GAPDH and CD14 interaction with antibody

To further validate GAPDH–CD14 interactions, phagocytosis of apoptotic J774 cells by THP-1 macrophages was carried out in the presence of CD-14 blocking antibody (Invitrogen #12-0149-42) CD14 and evaluated by flowcytometry.

### Statistical analysis

All experiments are repeated at least three times and statistical analysis was performed using unpaired Student’s *t* test. Flow Jo software was used to analyze the flow cytometry histograms and dot plots. Image J software is used to quantify the Western blots.

## Supplementary information

Supplementary Figures

Supplementary Table

## Data Availability

All data generated or analyzed during this study are included in this published article [and its supplementary information files].
