# Supplementary material for: Exposure of a specific pleioform of multifunctional glyceraldehyde 3-phosphate dehydrogenase initiates CD14-dependent clearance of apoptotic cells
Source: Cell Death Dis. 2021 Sep 30;12(10):892. doi: 10.1038/s41419-021-04168-8 (PMC8482365; doi:10.1038/s41419-021-04168-8)
Supplement: Supplementary file 1 — Supplementary Figures [file 41419_2021_4168_MOESM1_ESM.pdf]

<u>**Supplementary Material**</u>

Supplementary Figure S1.

(A) J774 cells were induced to undergo apoptosis using actinomycin D, live and apoptotic population of cells was revealed by double colour staining and flow cytometry based analysis. Cells were sorted through a 70 μm nozzle using BD FACS Aria™ III Sorter, a highly pure 2-way purity sorting was carried out and cells were collected in appropriate annexin binding buffer. Sorted cells were confirmed for purity. **(B)** In comparison to live cells (Annexin V- and 7AAD-) GAPDH level on surface of apoptotic cells (Annexin V+ and 7AAD-) was significantly higher. **(C)** The sorted cell populations ($1x10^6$ cells/sample) were then utilized for analysis of caspase activation assay as described in methods. Apoptotic cell population (Annexin V+ and 7AAD-) that exposed higher levels of GAPDH on their surface demonstrated significantly enhanced higher caspase activation as in comparison to live cells (Annexin V- and 7AAD-). ***$p<0.001$, n=3.

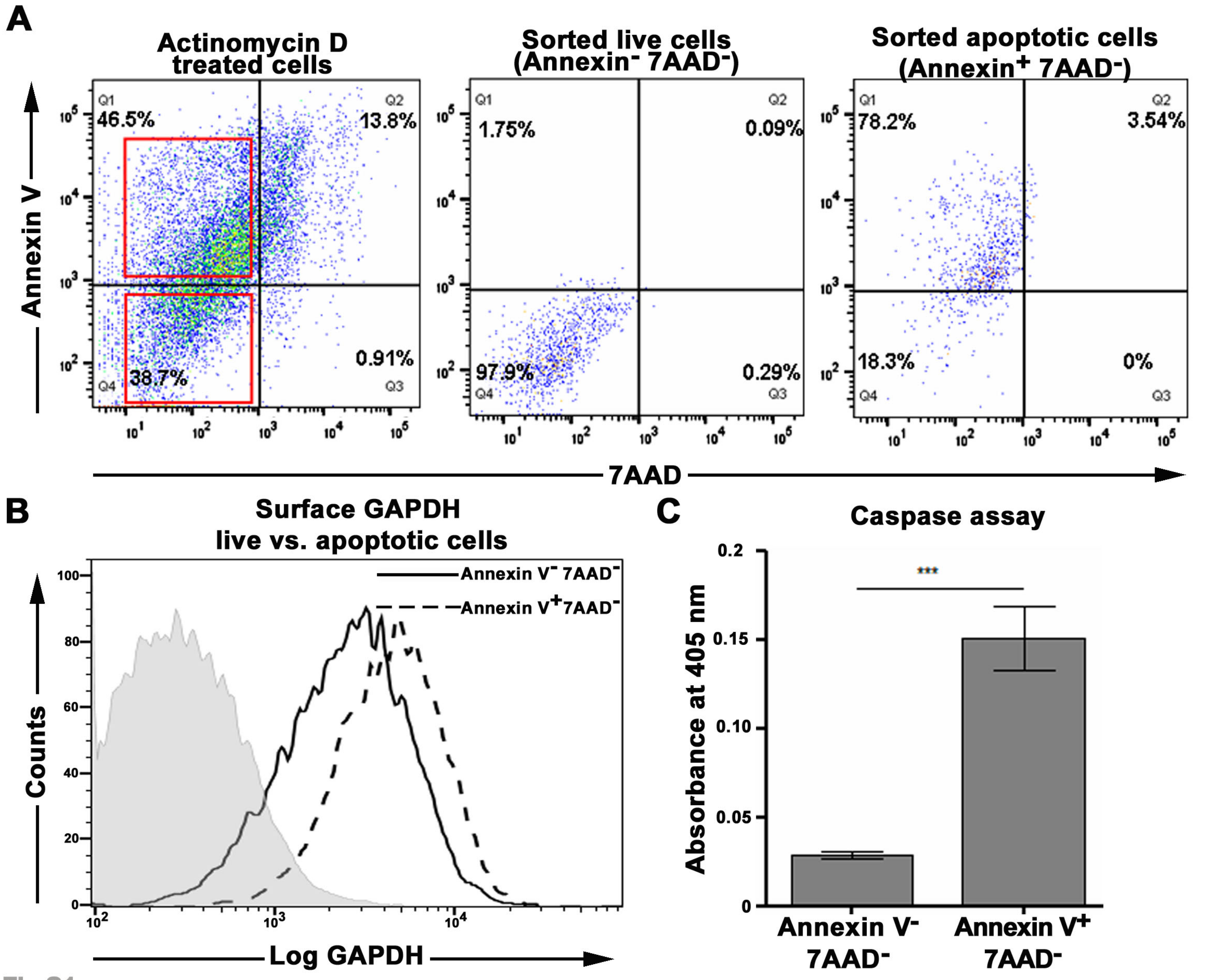

A
Actinomycin D treated cells
Sorted live cells (Annexin⁻ 7AAD⁻)
Sorted apoptotic cells (Annexin⁺ 7AAD⁻)
Annexin V
7AAD
Q1 46.5%
Q2 13.8%
38.7% Q4
0.91% Q3
Q1 1.75%
Q2 0.09%
97.9% Q4
0.29% Q3
Q1 78.2%
Q2 3.54%
18.3% Q4
0% Q3
B
Surface GAPDH live vs. apoptotic cells
Annexin V⁻ 7AAD⁻
Annexin V⁺7AAD⁻
Counts
Log GAPDH
C
Caspase assay
Absorbance at 405 nm
***
Annexin V⁻ 7AAD⁻
Annexin V⁺ 7AAD⁻

Fig.S1

Supplementary Figure S2.

(A) Flow cytometry reveals elevated GAPDH levels on surface of CHO-TRVb cells induced to undergo apoptosis by the intrinsic pathway using staurosporine. Histogram is representative from three independent experiments. The GAPDH recruited to apoptotic cell surface does not interact with either holo transferrin **(B)** or apo transferrin **(C)**. Both iron depleted as well as iron loaded cells demonstrate ~2 fold increase in surface GAPDH **(D)** along with a concomitant increase in cell surface binding by holo or apo forms of transferrin respectively. On the other hand in spite of ~7 fold increase in surface GAPDH exposure on CHO-TRVb apoptotic cells **(D)**, the binding by either forms of transferrin is practically unchanged **(B&C)**. All experiments were repeated three times. Data from representative experiment is presented. **(E)** Elevated GAPDH expression on surface of nutrient stressed but non apoptotic cells does not result in enhanced phagocytosis. Phagocytosis of iron depleted, iron loaded and apoptotic J774 cells by THP-1 derived macrophages was compared with phagocytosis of control live J774 cells. No significant increase in phagocytosis is observed when cell surface GAPDH recruitment is enhanced in cells due to modulation of cellular iron. Data is represented as the % of THP1 cells that engulf J774 cells ± SD. Inset the same data presented as % Phagocytosis taking phagocytosis of apoptotic J774 cells as 100%. All experiments were repeated three times ***$p<0.001$, n=3. **(F)** Decrease in apoptotic PS exposing J774 cell population after treatment with scramblase inhibitor. **(G)** Caspase assay reveals no effect of scramblase inhibitor on progression of apoptosis. **(H)** Double staining and flowcytometry based analysis reveals that the induction of apoptosis by actinomycin D in control empty vector and GAPDH K/D cell populations is comparable. **(I)** Exposure of GAPDH on surface of GAPDH K/D cells is significantly decreased and upon induction of apoptosis in these cells there is no significant increase in surface GAPDH. Graph is presented as MFI of GAPDH signal ± SEM. (***$P<.0001$, $n=10^4$ cells, representative of 3 independent experiments). **(J)** Overlay histogram to represent the significantly lower surface exposure of GAPDH on GAPDH K/D apoptotic cells as compared to empty vector apoptotic cells.

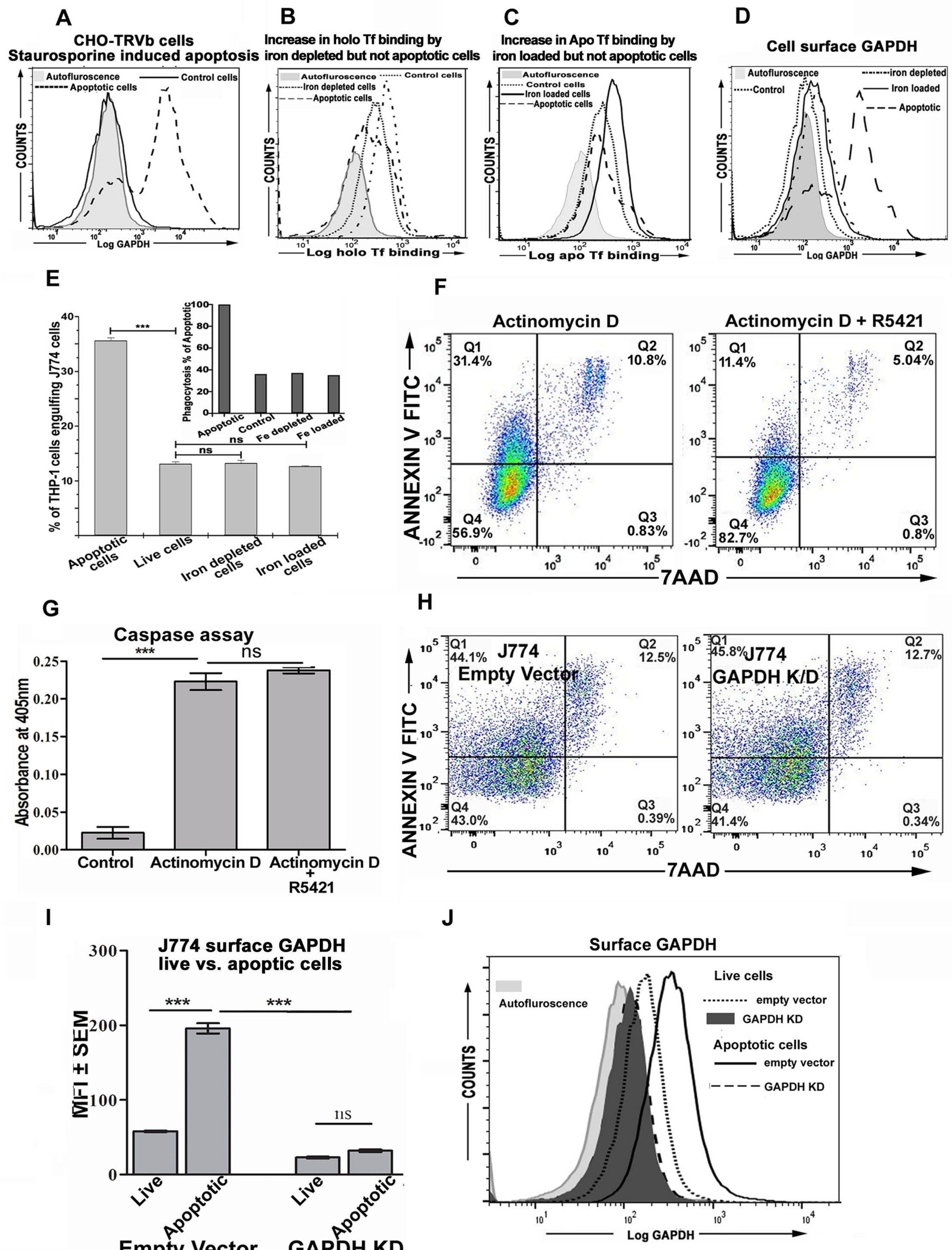
A
CHO-TRVb cells
Staurosporine induced apoptosis
Autofluroscence
Control cells
Apoptotic cells
COUNTS
Log GAPDH
B
Increase in holo Tf binding by
iron depleted but not apoptotic cells
Autofluroscence
Control cells
Iron depleted cells
Apoptotic cells
COUNTS
Log holo Tf binding
C
Increase in Apo Tf binding by
iron loaded but not apoptotic cells
Autofluroscence
Control cells
Iron loaded cells
Apoptotic cells
COUNTS
Log apo Tf binding
D
Cell surface GAPDH
Autofluroscence
iron depleted
Control
Iron loaded
Apoptotic
COUNTS
Log GAPDH
E
% of THP-1 cells engulfing J774 cells
Phagocytosis % of Apoptotic
Apoptotic
Control
Fe depleted
Fe loaded
***
ns
ns
Apoptotic cells
Live cells
Iron depleted cells
Iron loaded cells
F
Actinomycin D
Actinomycin D + R5421
Q1 31.4%
Q2 10.8%
Q4 56.9%
Q3 0.83%
Q1 11.4%
Q2 5.04%
Q4 82.7%
Q3 0.8%
ANNEXIN V FITC
7AAD
G
Caspase assay
***
ns
Absorbance at 405nm
Control
Actinomycin D
Actinomycin D + R5421
H
Q1 44.1%
J774 Empty Vector
Q2 12.5%
Q4 43.0%
Q3 0.39%
Q1 45.8%
J774 GAPDH K/D
Q2 12.7%
Q4 41.4%
Q3 0.34%
ANNEXIN V FITC
7AAD
I
J774 surface GAPDH
live vs. apoptic cells
***
***
ns
MFI ± SEM
Live
Apoptotic
Live
Apoptotic
Empty Vector
GAPDH KD
J
Surface GAPDH
Autofluroscence
Live cells
empty vector
GAPDH KD
Apoptotic cells
empty vector
GAPDH KD
COUNTS
Log GAPDH

Fig.S2

Supplementary Figure S3

(A) Co-Immunoprecepitation of live cell membrane fractions does not reveal any significant interaction between GAPDH on surface of live cells with CD14 on phagocyte membrane**.** Membrane fractions from live THP-1 J774 cells were prepared and used for co-ip as described in materials and methods. **(B)** Quantification of cell surface knock down of CD14 in THP-1 cells. Flow cytometry analysis reveals that CD14 on surface of cells is significantly reduced in KD cells. For staining, cells were incubated with FACS block and subsequently stained with human anti-CD14-APC antibody. Bar graph represents MFI of CD14 staining ± SEM. (***P<0.0001, n=10^4cells). **(C)** Flowcytometery based analysis also indicates that phagocytosis of apoptotic cells is dependent upon both, GAPDH on apoptotic cell surface and CD14 on phagocytes. Apoptotic J774 EV or GAPDH KD cells were labeled with Vybrant DiD dye. Separately THP-1 phagocytes EV or CD14 KD were labeled with CFSE. The **1st panel** (phagocytosis of live J774 cells) is shown as control where minimal phagocytosis is observed. When either GAPDH on apoptotic cells **(3nd panel)** or CD14 on phagocytes **(4th panel)** is decreased there is a significant reduction in the percentage of macrophages that ingest apoptotic cells. When both GAPDH on apoptotic cells and CD14 on macrophages is knocked down there is further decrease in ability of macrophages to internalize the apoptotic cells **(5th panel)**. Experiments were repeated three times and representative panels are presented. Data in bar graph **(D)** is % Phagocytosis ± SD taking phagocytosis of J774 empty vector apoptotic cells by THP-1 empty vector cells **(2nd panel)** as 100%. (***P<0.0001, n=3).

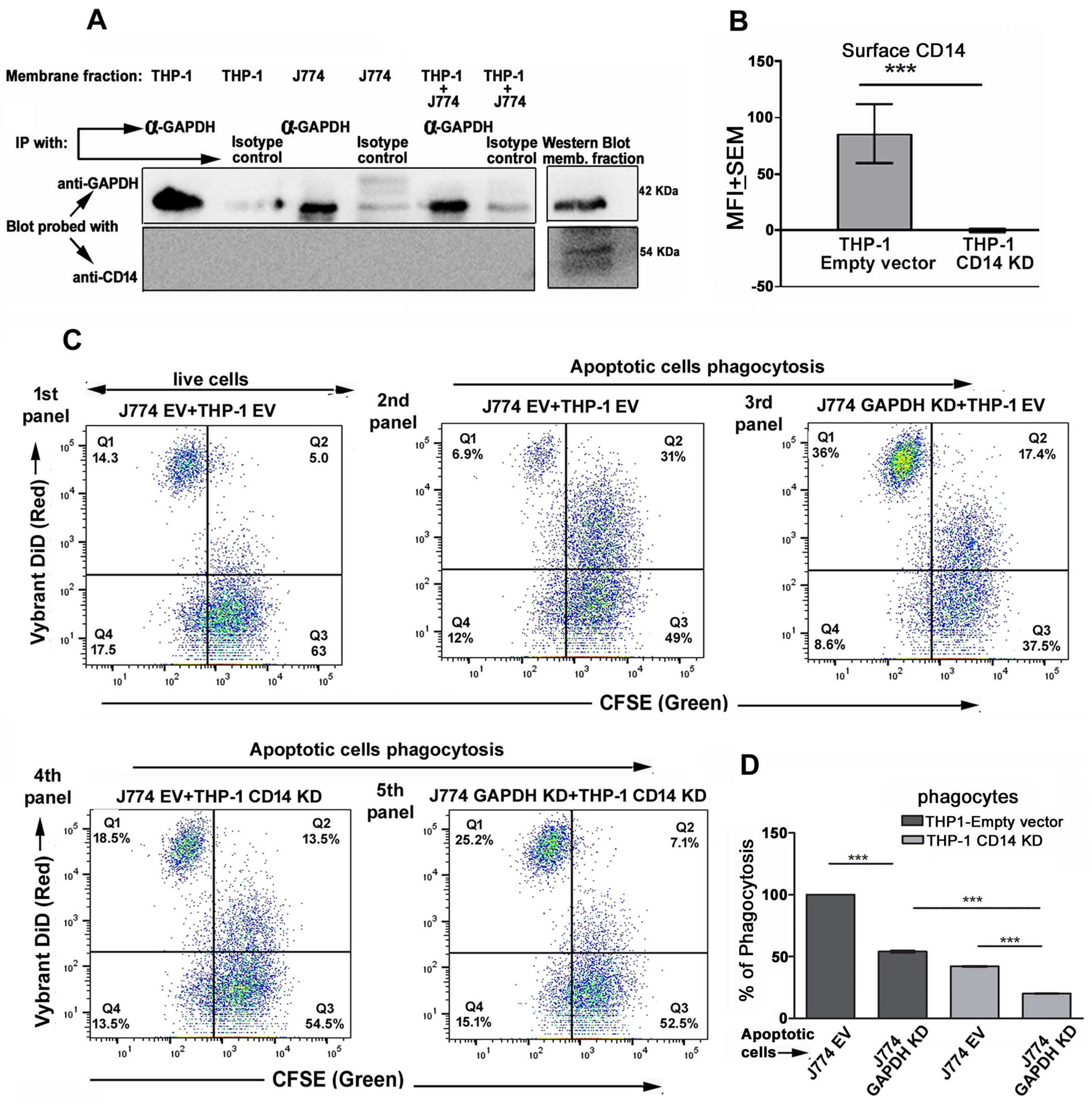
A
Membrane fraction: THP-1 THP-1 J774 J774 THP-1 + J774 THP-1 + J774
IP with:
α-GAPDH
Isotype control
α-GAPDH
Isotype control
α-GAPDH
Isotype control
Western Blot memb. fraction
anti-GAPDH
Blot probed with
anti-CD14
42 KDa
54 KDa
B
Surface CD14
***
MFI+SEM
THP-1 Empty vector
THP-1 CD14 KD
C
live cells
Apoptotic cells phagocytosis
1st panel
J774 EV+THP-1 EV
Q1 14.3
Q2 5.0
Q4 17.5
Q3 63
2nd panel
J774 EV+THP-1 EV
Q1 6.9%
Q2 31%
Q4 12%
Q3 49%
3rd panel
J774 GAPDH KD+THP-1 EV
Q1 36%
Q2 17.4%
Q4 8.6%
Q3 37.5%
Vybrant DiD (Red)
CFSE (Green)
Apoptotic cells phagocytosis
4th panel
J774 EV+THP-1 CD14 KD
Q1 18.5%
Q2 13.5%
Q4 13.5%
Q3 54.5%
5th panel
J774 GAPDH KD+THP-1 CD14 KD
Q1 25.2%
Q2 7.1%
Q4 15.1%
Q3 52.5%
Vybrant DiD (Red)
CFSE (Green)
D
phagocytes
THP1-Empty vector
THP-1 CD14 KD
% of Phagocytosis
***
***
***
Apoptotic cells
J774 EV
J774 GAPDH KD
J774 EV
J774 GAPDH KD

Fig.S3
