# Supplementary material for: Exposure of a specific pleioform of multifunctional glyceraldehyde 3-phosphate dehydrogenase initiates CD14-dependent clearance of apoptotic cells
Source: Cell Death Dis. 2021 Sep 30;12(10):892. doi: 10.1038/s41419-021-04168-8 (PMC8482365; doi:10.1038/s41419-021-04168-8)
Supplement: Supplementary file 2 — Supplementary Table [file 41419_2021_4168_MOESM2_ESM.doc]

**Supplementary Table 1**

**LC-MS/MS analysis of membrane-associated GAPDH from control and apoptotic cells**.

**A Membrane GAPDH from control cells**

1 MVKVGVNGFGRIGRLVTRAAIC(carbamido)SGK(dimethyl)VEIVA IN(dimethyl)D(methyl)PFID(methyl)LN Y(phos)MVYMFQ(deamid)Y(phos)D(methyl)ST(phos)

51 HGK(acetyl)FNGTVK(methyl)AENGK(dimethyl)LVINGK PITIFQERDP TNIKWGEAGA E(methyl)YVVES(methyl)TGVF

101 T(methyl/phos/GPI)T(methyl/phos)M(oxid)EK(palmitoyl)AGAHL K(dimethyl/GPI)GGAKRVIIS APSADAPMFVMGVN HE(methyl)K(methyl)YD(methyl)

N(dimethyl)S(methyl)LK(acetyl)IVS(phos)NAS(phos)C

151 TTNCLAPLAK VIHDNFGIVE GLMTTVHAIT AT(phos)Q(deamid)K(acetyl)TVD(methyl)GPS(phos) GK(GPI)LWRDGRGA

201 AQNIIPASTG AAKAVGKVIP ELNGKLTGMA FRVPTPNVS(methyl)VVDLT C RLEKP

251 AKYDDIKKVV KQASEGPLKG ILGYTEDQVV SCDFNSNSHS SaTFDAGAGIA

301 LNDNFVKLIS WYDNEYGYSN RVVDLMAYMA SKE

**B Membrane GAPDH from apoptotic cells**

1 MVKVGVNGFG RIGRLVTRAAIC(carbamido)S(phos)GK(acetyl)VE(methyl)IVAIN(deamid)DPFIDLNY(phos)MV(GPI)Y(phos)MFQ(deamid)YD(methyl)S

T(palmi)H(methyl/dimethyl)GK(GPI/succinyl)FN(dimethyl)GT(phos)VK(GPI/palmi/succinyl)A

51 EN(deamid)GK(myristoyl)LVIN(deamid)GK(adp-ribo) PITI(GPI)FQ(GPI)ERDP T(METHYL)NIK(ADP-ribo)WGE(methyl)AGA E(methyl)Y(phos)VVESTGVF

101 TTMEKAGAHL KGGAKRVIISAPSADAP(proline to pyro)MFV MGVNHE(methyl)K(acetyl)YDN SLKIVSNASC

151 TTNCLAPLAK VIHDNFGIVE GLMT(phos)TVH(methyl)AIT(methyl) ATQ(deamid)K(dimethyl/GPI)T(phos)VD(methyl)GP(proline to pyro)S(methyl) GK(palmi)LW

R(methyl/GPI)DGRGA

201 AQN(dimethyl)IIP(proline to pyro)AS(methyl)T(methyl/phos)G AAK(acetyl/ADP-ribco)AVGK(methyl/succinyl)VIP(proline to pyro) ELN(deamid)GK(acetyl/GPI)LTGMAFRVPTPNVS(METHYL)V VDLT C RLEKP

251 AK(ADP-ribo)YDDIKKV(methyl)V K(acetyl)Q(deamid)AS(phos)EGPLK(dimethyl/GPI)G ILGYTEDQVV SCDFNSNSHS STFDAGAGIA

301 LNDNFVK(acetyl)LIS WYD(methyl)NE(methyl)YGYSN R(dimethyl)VVD(methyl)LM(oxid)AYM(oxid)A SK(palmi)E(GPI)

C

| **PTMs** | **GAPDH (control)** | **GAPDH (Apoptosis)** |
| --- | --- | --- |
| **Methyaltion (D, E, S, T,K, V, H, R)** | **12[ D187, D48, E91, S96, T101, D33, D37, K59, K137, D139, S141, T102]** | **15[D313, E315, H51, K217, S208, E27, T209, H177, T180, S190, R195, D324, V259, T81, E87, E91]** |
| **Dimethylation (N, R, K, H)** | **4[N32, K25, K111, N140]** | **6[R321, H51, N203, K184, K269, N55]** |
| **Acetylation (K)** | **4[K53, K184, K64, K143]** | **6[K307, K225, K25, K213, K261, K137]** |
| **Phosphorylation (S,T,Y)** | **10[S190, Y47, T50, Y40, S146, S149, T182, T101, T102]** | **9[Y40,T209, T174, T185, Y43, S264, S123, Y92, T57]** |
| **Deamidation ( N,Q)** | **4[Q183, Q46]** | **7[N62, N68, N223, N32, Q183, Q46, Q262]** |
| **Oxidation (M, C)** | **1[M103]** | **2[M326,M329]** |
| **Carbamidomethyaltion (C)** | **1[C22]** | **2[C22,C245]** |
| **GPI anchor (protein C-term)** | **3 [K192, T101, K111]** | **10[K59, Q76, K225, I74, V42, K184, K269, R195, K53, E333]** |
| **Myristolyaltion (K, N-term G)** | **ND*** | **1[K64]** |
| **ADP ribosylation** | **ND*** | **4[K70,K213, K252, K84]** |
| **Proline oxidation to pyroglutamic (P)** | **ND*** | **4[P220, P206, P189, P127]** |
| **Palmitoylation (C,K,S,T)** | **[K105]** | **4[K192, T50, K332, K59]** |
| **Succinyl Lysine (K)** | **ND*** | **3[K59, K217, K53]** |
| **Farnesylation** | **ND*** | **ND*** |

J774 cells were induced to undergo apoptosis by actinomycin D and then membrane fractions from apoptoic and control cells were purified. Membrane proteins from both samples were extracted and subjected to 12% SDS-PAGE. Bands corresponding to GAPDH were excised and digested with trypsin. Peptide analysis by PEAK following LC-MS/MS confirmed proteins to be GAPDH. The protein confidence score was >100, with sequence coverage of >90% in both cases. **(A)** PTMs observed in GAPDH recruited to the cell membrane in control cells. **(B)** PTMs observed in GAPDH recruited to the cell membrane in apoptotic cells. **(C)** Comparative analysis of various PTMs and the corresponding modified residues in GAPDH recruited to the cell membrane under both conditions.
